# Metal–5-Fluorouracil–Histamine Complexes: Solution, Structural, and Antitumour Studies

**DOI:** 10.1155/MBD.2002.337

**Published:** 2002

**Authors:** Sadhna Tyagi, Sukh Mahendra Singh, Sujan Gencaslan, W. S. Sheldrick, Udai P. Singh

**Affiliations:** 1 Department of Chemistry Indian Institute of Technology Roorkee Roorkee 247 667 India; 2 School of Biotechnology Banaras Hindu University Varanasi 221 005 India; 3 Lehrstuhl für Analytische Chemie Ruhr-Universität Bochum Bochum D-44801 Germany

## Abstract

Solution studies were performed pH-metrically to study the interaction of Co(II), Ni(II), Cu(II), Zn(II) and Cd(II) metal ions with 5-fluorouracil (5FU) and histamine (Hm) separately (binary) and in the presence of each other (ternary) at 25±0.1^ °^C temperature and a constant ionic strength of 0.1 M NaNO_3_ in aqueous solution. The ternary complexes have been found to be more stable than the corresponding binary complexes as shown by the positive value of ΔlogK. The species distribution curves have been obtained using the computer programme BEST. On the basis of species distribution results, efforts were also made to prepare some mixed complexes of Co(II), Ni(II), Cu(II), Zn(II) and Cd(II) ions by performing the reaction of their metal nitrates, 5FU and Hm in aqueous ethanol medium at suitable pH. The isolated solid complexes were characterized by different physico-chemical method in order to suggest the possible binding site of the ligands and the structure of the resultant complexes. All these complexes were checked for their antitumour activity by injecting in Dalton's lymphoma (DL) and Sarcoma-180 (S-180) bearing C_3_H/He mice. The results indicate that some complexes have good antitumour activity both *in vivo* and *in vitro*.


### METAL–5-FLUOROURACIL–HISTAMINE COMPLEXES: SOLUTION, STRUCTURAL, AND ANTITUMOUR STUDIES

Sadhna Tyagi[1], Sukh Mahendra Singh[2], Sujan Gencaslan[3], W.S. Sheldrick[3] and Udai P. Singh[1]*

[1] Department of Chemistry, Indian Institute of Technology Roorkee, Roorkee - 247 667, India
[2] School of Biotechnology, Banaras Hindu University, Varanasi - 221 005, India
[3] Lehrstuhl für Analytische Chemie, Ruhr-Universität Bochum, D-44801 Bochum, Germany

### ABSTRACT

Solution studies were performed pH-metrically to study the interaction of Co(II), Ni(II), Cu(II), Zn(II) and Cd(II) metal ions with 5-fluorouracil (5FU) and histamine (Hm) separately (binary) and in the presence of each other (ternary) at 25 ± 0.1 °C temperature and a constant ionic strength of 0.1 M $NaNO_3$ in aqueous solution. The ternary complexes have been found to be more stable than the corresponding binary complexes as shown by the positive value of ΔlogK. The species distribution curves have been obtained using the computer programme BEST. On the basis of species distribution results, efforts were also made to prepare some mixed complexes of Co(II), Ni(II), Cu(II), Zn(II) and Cd(II) ions by performing the reaction of their metal nitrates, 5FU and Hm in aqueous ethanol medium at suitable pH. The isolated solid complexes were characterized by different physico-chemical method in order to suggest the possible binding site of the ligands and the structure of the resultant complexes. All these complexes were checked for their antitumour activity by injecting in Dalton's lymphoma (DL) and Sarcoma-180 (S-180) bearing $C_3H/He$ mice. The results indicate that some complexes have good antitumour activity both *in vivo* and *in vitro*.

### INTRODUCTION

5-Fluorouracil (5FU), a mono fluorinated product of uracil, has entirely different biological properties than uracil [1]. Since the time of its synthesis, 5FU has been increasingly employed alone or in combination with other cytotoxic drugs and hormones in the medical treatment of solid tumours. It has also been used in the treatment of breast, lung, ovary and cervix carcinomas [2]. The antitumour properties of 5FU against different tumour systems has also been found to be significantly enhanced by the co-administration of guanosine (in any combination) resulting in therapeutic synergism [3]. The mechanism of action of 5FU is not well known. In 1986, Joshi et al. [4] suggested that the 5FU is anabolised to 5-fluoro-2′-dexoyuridylic acid, a potent competitive inhibitor of thymidylate synthetase, and the enzyme which normally converts 2′-deoxyuridylic acid to thymidylic acid as essential component of DNA. This is due to the presence of fluorine atom at the critical C-5 position. Since 5FU and its anabolites are concentrated in cancer cells, this enzymatic blockade inhibits tumour growth by causing thymineless death of neoplastic cells. Histamine (Hm), a decarboxylated product of histidine, is a potent vasodilator and is released in certain tissue as a result of allergic hypersensitivity or inflammation. Histamine also plays an important role in diseases.

The antitumour properties of 5FU and scanty information on its metal complexes in combination with histamine as antitumour agent encouraged to undertake solution, solid and antitumour activity studies of 5FU-Histamine mixed complexes.

Although several workers have reported the antitumor properties of 5FU and its metal complexes [5], also the solution studies on 5FU [6] and histamine separately [7], the work on the chemotherapeutic properties of 5FU-histamine metal complexes is not available in the literature.

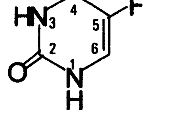

5-Fluorouracil

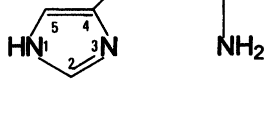

Histamine

Fig. 1. Structures of 5-fluorouracil and histamine.

The present paper reports the solution, structural and antitumour studies of Co(II), Ni(II), Cu(II), Zn(II) and Cd(II) complexes with 5-fluorouracil (5FU) as one ligand, and histamine (Hm) as another ligand.

### RESULT AND DISCUSSION

### Solution studies

The pH-metric titration curves (Figure 2a-e) were plotted as pH *vs.* a (where a is the number of moles of alkali required per mole of the ligand) for 5FU (curve a), M-5FU (curve b), Hm (curve c), M-Hm (curve d) and M-5FU-Hm (curve e) systems. The deprotonation constant values ($pK_n$) and various formation ccnstants for binary and ternary systems (Table 1) were calculated by using the literature method [8] and the computer programs pKAS and BEST [9] respectively. From the ligand curves a and c, the first proton dissociation constant ($pK_1$) values for 5FU and Hm are calculated to be 7.55±0.06 and 6.20±0.06 by considering the first deprotonation equilibrium $H_2L$ == $H^+$ + $HL^-$ and computer program pKAS [9]. Similarly, the second deprotonation $HL^-$ == $H^+$ + $L^{-2}$ constant ($pK_2$) values are also evaluated to be 10.60±0.03 and 9.85±0.04 for the two ligands, respectively.

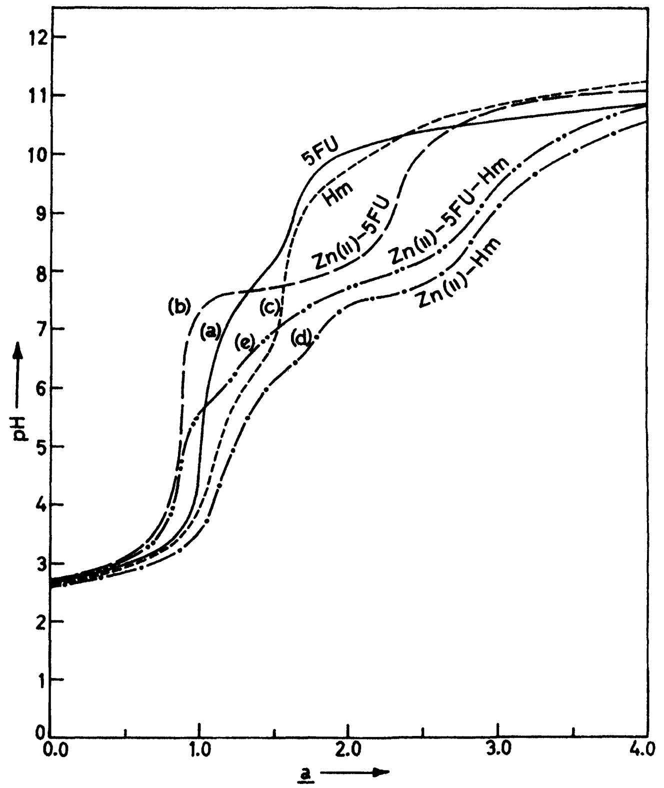

Fig. 2. pH metric titration curves of (a) 5-fluorouracil solution ( 1.0 mM), (b) metal ion (1.0 mM) + [a], (c) histamine (1.0 mM), (d) metal ion (1.0 mM) + [c], (e) metal ion (1.0 mM) + [a] + [c] at T = 25 ± 0.1 °C and $\mu$ = 0.1 M ($NaNO_3$) in aqueous solution. Where a is number of mole of alkali per mole of ligand.

In Fig. 2, the titration curves b and d account for the association of metal ions with 5FU (curve b) and Hm (curve d), respectively. The overall stability constant for each binary system, M-5FU as well as M-Hm was evaluated by using the method described earlier [8] and the BEST computer programme [9].

The order of stability of 1:1 binary system of 5FU and Hm is Co(II)<Ni(II)<Cu(II)>Zn(II)>Cd(II), which is in conformity with Irving-William's order. Although the Cu(II) complexes should have higher stability as compare to the Co(II) and Ni(II) complexes but it has been found to be unusually higher than could be expected from the ionic radii and electronegativity considerations. It may be attributed to the unique electronic configuration ($d^9$) of the Cu(II) ion which is capable of additional stabilization due to the Jahn-Teller distortion [10]. In addition to this, the Hm molecule is supposed to be the more basic than 5FU, hence M(II)-Hm systems should be more stable than the corresponding M(II)-5FU systems, which is also supported from the results presented in Table 1. It may be due to the binding nature of Hm as it binds with metal ions through both the imidazole and amine groups produce a chelate ring that enhances the stability of metal complexes of Hm. The titration curve clearly exhibits the interaction of 5FU and Hm with metal ions in the presence of each other. The stability constants of various ternary metal-ligand systems were evaluated by using a previous method [8] and the BEST computer programme [9] (Table 1). The overall stability constants of the binary and the corresponding ternary metal-ligand complexes were compared and it has been found that the ternary complexes are more stable than the metal-5FU complexes but are less stable than the corresponding metal-Hm complexes. It may be due to higher concentration of the electrons around the $[M\text{-}5FU]^{+}$ system in comparison to the $[M(H_2O)_n]^{2+}$ system hence statistical steric and electrostatic factors lead to lower stability constants of ternary system than the binary system of Hm in solution.

**Table 1.** Overall stability constants (log K)[a] of binary (1:1) and ternary (1:1:1) metal-ligand complexes formed in aqueous solution at T = 25 ± 0.1 °C and $\mu$ = 0.1 M $NaNO_3$

| Metal Ions | M:5FU $\log K^{M}_{MA}$ | | M:Hm $\log K^{M}_{ML}$ | | M:5FU:Hm $\log K^{MA}_{MAL}$ | | ΔlogK | |
|---|---|---|---|---|---|---|---|---|
| | (a) | (b) | (a) | (b) | (a) | (b) | (a) | (b) |
| Co(II) | 5.08±0.15 | 5.91 | 6.21±0.15 | 8.37 | 5.31±0.03 | 5.25 | -0.90 | -3.12 |
| Ni(II) | 5.91±0.06 | 5.32 | 7.38±0.09 | 8.86 | 5.99±0.06 | 5.44 | -1.39 | -3.42 |
| Cu(II) | 8.12±0.07 | 8.22 | 9.74±0.14 | 10.24 | 8.28±0.07 | 7.88 | -1.46 | -2.36 |
| Zn(II) | 6.11±0.15 | 8.01 | 6.43±0.17 | 8.32 | 6.32±0.14 | 6.23 | -0.11 | -2.09 |
| Cd(II) | 5.76±0.09 | 6.02 | 6.02±0.13 | 7.90 | 5.93±0.09 | 5.85 | -0.09 | -2.05 |

[a]Deviation (3σ) range in between ±0.06 to 0.18.

Method (a) Interpolation of Half n values; (b) Average value method

ΔlogK: Difference between the stability constant of ternary complex and binary complex of secondary ligand.

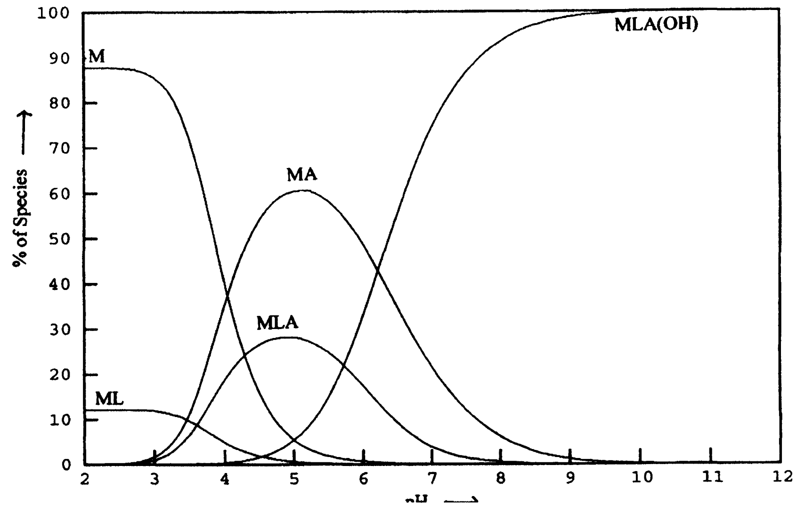

Fig. 3. Species distribution curve of Zn(II):5-fluorouracil:histamine (1:1:1) ternary system.

The species distribution for various possible species in solution has been performed for all the metal ions reported in the present work and the species distribution plot for Zn(II)-5FU-Hm has been given in Fig. 3 as representative graph. Fig. 3 indicates the presence of free metal and 1:1 neutral complex of M-5FU in solution around pH 2.0. Their concentration start decreases with increase of the concentration of neutral species of M-Hm and M-5FU-Hm and attain almost a zero value at higher pH range. From the species distribution curve it can be stated further that the hydroxo species of ternary complex predominate the neutral species (i.e. 100% at pH 10.0) being formed comparatively in small amounts in the lower pH range under investigation. Again formation of (1:1) neutral species suggests the bidentate nature of both the ligand in binary system. Same pattern of species distribution occurs in the ternary system of other metal ions. At pH 2.0, free metals are present in 73% to 91% amount. Association of 5FU with each metal ions start at very low pH range (~ 2.0) resulting in the formation of M-5FU species in which metal ions distributed as: ~ 12% for Co(II), Cu(II) and Zn(II); ~ 8.5% in Cd(II) and ~ 24% in Ni(II).

**Table 2.** Analytical data of the complexes

| | | Amount Found (Calc.) (%) | | | | |
|---|---|---|---|---|---|---|
| Complex | Color | M | C | H | N | Melting Point (°C) |
| Co(5FU)(Hm)(OH).2$H_2O$ | Light Pink | 16.55 (16.69) | 30.91 (30.61) | 03.69 (03.42) | 20.05 (19.80) | >300 |
| Ni(5FU)(Hm)(OH).2$H_2O$ | Green | 16.54 (16.64) | 30.04 (30.63) | 03.36 (03.43) | 19.37 (19.84) | 290 |
| Cu(5FU)(Hm)(OH).2$H_2O$ | Intense Green | 17.63 (17.76) | 30.47 (30.22) | 03.48 (03.38) | 19.67 (19.58) | 180 |
| Zn(5FU)(Hm)(OH).2$H_2O$ | White | 18.05 (18.18) | 30.48 (30.06) | 03.42 (03.36) | 19.55 (19.48) | >300 |
| Cd(5FU)(Hm)(OH).2$H_2O$ | White | 28.81 (27.65) | 27.76 (26.59) | 03.03 (02.97) | 17.42 (17.22) | >300 |

Furthermore association of Hm with each metal ions and M-5FU start at pH range (i.e. ~ 3.0) resulting in the formation of M-Hm and M-5FU-Hm species are as: ~ 61% - 72% for Cd(II), Co(II) and Zn(II) at pH ~ 5.0; ~ 78% for Cu(II) at pH ~ 4.0; ~ 46% for Ni(II) at pH ~ 5.0 and ~ 11% - 28% for Cd(II), Co(II) and Zn(II) at pH ~ 5.0; ~ 13% for Cu(II) at pH 3.0; ~ 38% for Ni(II) at pH 4.0 respectively. Finally the total metal distributed into ML, MA and MLA species get converted into the hydroxo species ($MLA(OH)^-$) at higher pH (i.e. > 8.0) and the metal ion distribution observed in about 100% at the corresponding pH. Again species distribution data clearly show that the ternary complexes are less stable than binary complexes of secondary ligand.

All the isolated mixed ligand complexes are colored except those of Zn(II) and Cd(II) and involve 1:1:1 metal to 5FU to Hm ratio (where M = Co(II), Ni(II), Cu(II), Zn(II) or Cd(II)). Most of the complexes do not melt up to 300 °C as reported in Table 2.

**Infrared studies**

The infrared spectrum of 5FU is reported in the literature [5a] and IR band assignments for histamine have been made by comparing its spectrum with various amino acids reported in the literature [11-13]. Table 3 records some important infrared data for the ligands and their mixed complexes. Histamine molecule is potentially a bidentate and forms complex with bivalent cations through the imidazole nitrogen and the amino nitrogen. The neutral imidazolyl group of Hm exists in tautomeric equilibrium between $N_1$-protonated form and the $N_3$-protonated one. Either one of the unprotonated imidazole nitrogens of these tautomers can participate in coordination to metal ions. Basically histamine molecule has three coordinating sites viz. $N_1$, $N_3$ imidazole nitrogen and amino nitrogen. As reported in the literature [11-13], it is sterically impossible for a Hm molecule to make a chelate ring with metal ions through $N_1$ atom and amino nitrogen. This means that when Hm molecule will coordinate through $N_1$ imidazole atom, its amino group will exist in the free and $NH_3^+$ state. The imidazole nitrogen of Hm can also form hydrogen bonds. Infrared spectra of the complexes (Table 3) show shift in $NH_3^+$ bands of Hm (3110 $cm^{-1}$) either toward lower frequency side or toward higher frequency side on coordination. The shifting of νN-H band as compared to that in free Hm (3310 $cm^{-1}$) suggest the coordination of Hm through the nitrogen of amino group. This is further supported by the shift in the ν$C_7$-N band appearing at 1036 $cm^{-1}$ in free Hm.

The band observed at 1570 $cm^{-1}$ and 1252 $cm^{-1}$ in the spectrum of Hm can be assigned to the imidazole ring vibration of Hm for $N_3$ and amino nitrogen. On coordination the imidazole ring vibration shifts toward lower or higher frequency sides (Table 3), suggesting the coordination of Hm of the metal ions through its $N_3$ atom and amino nitrogen [14]. The bands at 1598 $cm^{-1}$ in the spectrum of free Hm is assigned to the ring vibration of the imidazolyl group due to the $N_1$ atom. This band in all complexes remains almost unchanged. Thus it may be concluded that in all these mixed complexes the Hm behaves as bidentate ligand coordinating to the central ion through its $N_3$ atom and amino nitrogen. There are several possible binding sites in 5FU viz. $C_2$=O, $C_4$=O, $N_1$-H and $N_3$-H groups. Table 3 shows significant change in the frequency of ν$N_3$-H band in the complexes indicating that $N_3$-H group takes part in coordination with metal ions in complex formation. In all complexes, a medium to strong metal-nitrogen stretching band appears in the range 242-260 $cm^{-1}$ [15, 16], indicating six coordination number around these metal ions. All of the mixed complexes exhibit νO-H (aquo) bands at 3500-3250 $cm^{-1}$ suggesting the presence of water molecules in the complexes [17]. The presence of

νM-O (aquo) band in lower region of infrared spectrum and absence of HOH bending bands due to lattice water in 1630-1600 $cm^{-1}$ region favour the coordinated nature of water molecules in these complexes [18].

**Table 3.** Relevant infrared data for ligands and their mixed complexes ($Cm^{-1}$)

| Band Assignment | Histamine | 5FU | Co(II) | Ni(II) | Cu(II) | Zn(II) | Cd(II) |
|---|---|---|---|---|---|---|---|
| νO-H | | - | 3410w | 3415w | 3420w | 3338w | 3440w |
| νN-H | 3310m | - | 3330m | 3328m | 3290m | 3260w | 3338w |
| νN-H | - | 3160m | 3142w | 3145w | 3180w | 3185w | 3140w |
| $\nu NH_3^+$ | 3110w,b | - | 3135w | 3130w | 3130w | 3137m | 3130w |
| νC=C in phase νC=C of imidazole ($N_1$)ring | 1596s,b | - | 1605s | 1595m | 1592s | 1598s | 1590s |
| νC=C of imidazole ($N_3$)ring | 1570m | - | 1555m | 1546m | 1555w | 1550w | 1540s |
| $\delta N_3$-H | - | 1430s | 1426w | 1436m | 1436m | 1422w | 1435s |
| Ring breathing for amino nitrogen | 1252s | - | 1274m | 1273w | 1275m | 1266s | 1270m |
| νCα-N | 1036s,b | - | 1054m | 1024m | 1018m | 1020w | 1054s |
| νM-O(aquo) | - | - | 408m | 408m | 474m | 368m | 355m |
| νM-N | - | - | 260m | 260m | 258m | 242w | 245m |

**Electronic spectral and Magnetic studies**

The magnetic moments of Cu(II) complex (Table 4) shows the presence of one unpaired electron. The d-d transition bands appearing in the region 600-900 nm for Cu(II) complex, favours distorted octahedral geometry around Cu(II) ion in the complex [19]. As reported in the literature that Co(II) high spin octahedral complexes have magnetic moment ranging from 4.7 to 5.2 B.M. and tetrahedral complexes have magnetic moment range 4.4 to 4.8 B.M. whereas Ni(II) octahedral complexes have magnetic moment 2.9 to 3.4 B.M. and tetrahedral complexes have magnetic moment 3.5 to 4.2 B.M. [20]. The $\mu_{eff}$ values and position of electronic spectral bands of present Co(II) and Ni(II) complexes as shown in Table 4 suggest octahedral geometry for Co(II)-5FU-Hm and Ni(II)-5FU-Hm complexes [19, 21].

**Table 4.** Solid state (Nujol-Mull) electronic spectral and magnetic moment data (305.5 K) of metal complexes.

| Complexes | $\lambda_{max}$ (nm) | $\mu_{eff}$ (B.M.) |
|---|---|---|
| $Co(5FU)(Hm)(OH).2H_2O$ | 550, 830 | 4.95 |
| $Ni(5FU)(Hm)(OH).2H_2O$ | 395, 670 | 3.21 |
| $Cu(5FU)(Hm)(OH).2H_2O$ | 610 | 2.01 |

**X-ray diffraction studies**

X-ray diffraction data (Table 5) of the complexes were indexed according to the method of Ito [22]. The indexing pattern yields the lattice constants a = 7.32 Å, b = 7.01 Å, and c = 6.79 Å for Zn(II) complex indicating orthorhombic symmetry for this complex.

On the basis of above spectroscopic studies, the octahedral structure has been proposed for all these complexes.

**Antitumour activity studies**

It has been observed that the ligand 5FU has significant antitumour activity with T/C value 136 at 12.5, 150 at 25.0 and 154 at 50.0 mg/kg body weight against Dalton's lymphoma tumour system. Among the ternary complexes of histamine, Co(II)-5FU-Hm and Zn(II)-5FU-Hm have pronounced antitumour activity with T/C values more than 125 at all the reported doses (Table 6). All the experimental mice treated with Zn(II)-5FU-Hm complex survived beyond six month at the dose of 50.0 mg/kg body weight. The other compounds are not effective against Dalton's lymphoma tumour system at these doses. The Table 7 shows the result obtained for antitumour activity for 5FU and its mixed complexes against sarcoma-180 test system *in vivo*. As shown in the Table 7, Co(II)-5FU-Hm and Zn(II)-5FU-Hm exhibit significant antitumour activity, having a T/C value >125 at all these reported doses. Similar results have been inferred regarding the significant therapeutic effect of the tested compounds from their % ILS values as shown in Table 6 and 7.

**Table 5.** X-Ray data of the complexes

| Powder Pattern line | 2θ | $d_{values}$ | Relative Intensity | $Q_{(calc)}$ | $Q_{(obs.)}$ | hkl |
|---|---|---|---|---|---|---|
| 1. | 2. | 3. | 4. | 5. | 6. | 7. |
| | | | $Co(5FU)(Hm)(OH).2H_2O$ Amorphous | | | |
| | | | $Ni(5FU)(Hm)(OH).2H_2O$ Amorphous | | | |
| | | | $Cu(5FU)(Hm)(OH).2H_2O$ Amorphous | | | |
| | | | $Zn(5FU)(Hm)(OH).2H_2O$ | | | |
| 1. | 12.089 | 7.3212 | 164 | 0.0186 | 0.0186 | 100 |
| 2. | 12.631 | 7.0079 | 294 | 0.0203 | 0.0203 | 010 |
| 3. | 13.026 | 6.7963 | 402 | 0.0216 | 0.0216 | 001 |
| 4. | 21.391 | 4.1538 | 346 | 0.0579 | 0.0592 | 120 |
| 5. | 22.451 | 3.9601 | 100 | 0.0637 | 0.0618 | 102 |
| 6. | 25.003 | 3.5614 | 102 | 0.0788 | 0.0774 | 301 |
| 7. | 26.227 | 3.3978 | 225 | 0.0866 | 0.0851 | 013 |
| 8. | 27.800 | 3.2090 | 168 | 0.0971 | 0.0977 | 311 |
| 9. | 29.153 | 3.0631 | 72 | 0.1065 | 0.1080 | 005 |
| 10. | 30.938 | 2.8903 | 247 | 0.1197 | 0.1210 | 222 |
| 11. | 31.555 | 2.8352 | 101 | 0.1244 | 0.1253 | 114 |
| 12. | 33.309 | 2.6898 | 82 | 0.1382 | 0.1387 | 250 |
| 13. | 34.331 | 2.6120 | 88 | 0.1465 | 0.1473 | 034 |
| 14. | 34.839 | 2.5751 | 102 | 0.1508 | 0.1522 | 620 |
| 15. | 37.367 | 2.4065 | 95 | 0.1726 | 0.1728 | 008 |
| 16. | 38.351 | 2.3470 | 87 | 0.1815 | 0.1823 | 171 |
| 17. | 42.404 | 2.1316 | 100 | 0.2200 | 0.2199 | 290 |
| 18 | 46.623 | 1.9480 | 47 | 0.2635 | 0.2640 | 364 |
| | | | $Cd(5FU)(Hm)(OH).2H_2O$ Amorphous | | | |

Ligand 5FU and its mixed complexes were also tested for their inhibitory effect on $^3$H-thymidine incorporation in Dalton's lymphoma, Sarcoma-180 and L-929 tumour cell *in vitro* at 5μg/ml, 10μg/ml and 20μg/ml doses. It is observed that most of those compounds which caused inhibition of $^3$H-thymidine incorporation with Dalton's lymphoma, Sarcoma-180 and L-929 tumour cells, also showed antitumour activity *in vitro* (Table 8). The other compounds which were found ineffective antitumour agent *in vivo* were also tested but they were found to have no inhibitory effects *in vitro* also, hence their results are not shown in the Table 8. It is evident from results obtained *in vitro* that there is a dose dependent inhibition of $^3$H-thymidine incorporation, 20 μg/ml doses of most of the compound was found to be most effective. The mechanism of antitumour action of these compounds is not well known. The present results suggest that the antitumour properties of these compounds may be due to their inhibitory action on the replication of DNA in tumour cells.

### EXPERIMENTAL

### Material and Methods

All chemicals were used of analytical grade. The metal nitrates used were from E. Merck grade. 5FU and histamine were obtained from Aldrich and Sigma Chemical Co. U.S.A respectively. $^3$H-Thymidine was obtained from Bhabha Atomic Research Centre, Mumbai, India. The tissue culture medium RPMI-1640 and the other reagents were purchased from Sigma Chemical Company (St. Louis, Mo. USA). All culture media were supplemented with 20 μg/mL gentamycin, 100 μg/mL streptomycin, 100 μg/mL penicillin and 10% heat-inactivated fetal calf serum (Biological Industries, Haemak, Israel). All test compounds were suspended in phosphate buffered saline solution (PBS) (pH 7.0).

**Table 6.** Screening data of mixed ligand complexes for antitumour activity against Dalton's lymphoma *in vivo*

| Compounds | Dosage ip injection mg/kg body weight[a] | Mean Lifespan of non survivors T/C (days)[b] | No. of mice Surviving >6 months | T/C % | % ILS |
|---|---|---|---|---|---|
| 5FU | 12.5 | 30/22 | - | 136.36 | 36.36 |
| | 25.0 | 33/22 | - | 150.00 | 50.00 |
| | 50.0 | 34/22 | - | 154.54 | 54.54 |
| Co(5FU)(Hm)(OH).2$H_2O$ | 12.5 | 38/22 | - | 172.72 | 72.72 |
| | 25.0 | 35/22 | - | 159.09 | 59.09 |
| | 50.0 | 36/22 | - | 163.36 | 63.63 |
| Ni(5FU)(Hm)(OH).2$H_2O$ | 12.5 | 25/22 | - | 113.63 | 13.63 |
| | 25.0 | 20/22 | - | 90.90 | - |
| | 50.0 | 24/22 | - | 109.09 | 09.09 |
| Cu(5FU)(Hm)(OH).2$H_2O$ | 12.5 | 16/22 | - | 72.72 | - |
| | 25.0 | 08/22 | - | 36.36 | - |
| | 50.0 | 13/22 | - | 59.09 | - |
| Zn(5FU)(Hm)(OH).2$H_2O$ | 12.5 | 35/22 | - | 159.09 | 59.09 |
| | 25.0 | 33/22 | - | 150.00 | 50.00 |
| | 50.0 | all alive | 6(100) | - | - |
| Cd(5FU)(Hm)(OH).2$H_2O$ | 12.5 | 19/22 | - | 86.36 | - |
| | 25.0 | 14/22 | - | 63.63 | - |
| | 50.0 | 03/22 | - | 13.63 | - |

T= tumoured, C = control; ILS = increased lifespan
[a]A single ip injection of the reported dose was given to six mice in each experiment.
[b]In calculating average survival time, mice surviving >6 months were not included.

**Table 7.** Screening data of mixed ligand complexes for antitumour activity against Sarcoma-180 *in vivo*

| Compounds | Dosage ip injection mg/kg body weight[a] | Mean Lifespan of non survivors T/C (days)[b] | No. of mice Surviving >6 months | T/C % | % ILS |
|---|---|---|---|---|---|
| 5FU | 12.5 | 13/7 | - | 185.7 | 85.7 |
| | 25.0 | 11/7 | - | 157.1 | 57.1 |
| | 50.0 | all alive | 6(100) | - | - |
| Co(5FU)(Hm)(OH).2$H_2O$ | 12.5 | 10/7 | - | 142.8 | 42.8 |
| | 25.0 | 12/7 | - | 171.4 | 71.4 |
| | 50.0 | 18/7 | - | 257.1 | 157.1 |
| Ni(5FU)(Hm)(OH).2$H_2O$ | 12.5 | 08/7 | - | 114.2 | 14.2 |
| | 25.0 | 06/7 | - | 85.7 | - |
| | 50.0 | 05/7 | - | 71.4 | - |
| | 12.5 | 07/7 | - | 100.0 | - |
| Cu(5FU)(Hm)(OH).2$H_2O$ | 25.0 | 08/7 | - | 114.2 | 14.2 |
| | 50.0 | 06/7 | - | 85.7 | - |
| | 12.5 | 17/7 | - | 242.8 | 142.8 |
| Zn(5FU)(Hm)(OH).2$H_2O$ | 25.0 | 10/7 | - | 142.8 | 42.8 |
| | 50.0 | 13/7 | - | 185.7 | 85.7 |
| | 12.5 | 04/7 | - | 57.1 | - |
| Cd(5FU)(Hm)(OH).2$H_2O$ | 25.0 | 03/7 | - | 42.8 | - |
| | 50.0 | 02/7 | - | 28.5 | - |

T = tumoured, C = control; ILS = increased lifespan
[a]A single ip injection of the reported dose was given to six mice in each experiment.
[b]In calculating average survival time, mice surviving >6 months were not included.

**Table 8.** Percentage inhibition of $^3$H-Thymidine incorporation in Dalton's lymphoma, Sarcoma-180 and L-929 Tumor Cell *in vitro**

| Compound | Dose 5μg/ml | 10μg/ml | 20μg/ml |
|---|---|---|---|
| | | **DL** | |
| Co(5FU)(Hm)(OH).2$H_2O$ | 50.36 | 48.04 | 29.02 |
| Zn(5FU)(Hm)(OH).2$H_2O$ | 92.15 | 93.24 | 95.28 |
| | | **S-180** | |
| 5FU | 47.36 | 48.08 | 57.80 |
| Co(5FU)(Hm)(OH).2$H_2O$ | 23.75 | 50.65 | 92.29 |
| Zn(5FU)(Hm)(OH).2$H_2O$ | 36.32 | 54.79 | 85.85 |
| | | **L-929** | |
| 5FU | - | 18.33 | 49.93 |
| Co(5FU)(Hm)(OH).2$H_2O$ | - | 64.71 | 65.53 |
| Zn(5FU)(Hm)(OH).2$H_2O$ | - | 30.27 | 32.15 |

*This table shows the results obtained for the compounds which show significant inhibition.

**Potentiometric pH Titration**

Solutions of Co(II), Ni(II), Cu(II), Zn(II) and Cd(II) nitrates were prepared in double distilled water and standardized by EDTA titration method [23]. Ligand solutions of 0.01 M 5FU and 0.01 M Hm were also prepared in double distilled water. Stocks solutions of sodium nitrate (1.0 M) and standard nitric acid (0.02 M) were used. Carbonate-free NaOH (0.2 M) solution was standardized against oxalic acid solution and used as titrant.

All the measurements were carried out at 25 $\pm$ 0.1°C using Schott CG841 pH-meter. The six mixtures A, B, C, D, E and F were prepared and titrated separately against 0.2 M NaOH ($CO_2$-free) bubbling $N_2$ in the cell during the titration: (A) $HNO_3$ (0.02 M, 5.0 ml) + $NaNO_3$ (1.0 M, 5.0 ml) + water; (B) $HNO_3$ (0.02 M, 5.0 ml) + $NaNO_3$ (1.0 M, 5.0 ml) + 5FU (0.01 M, 5.0 ml) + water; (C) $HNO_3$ (0.02 M, 5.0 ml) + $NaNO_3$ (1.0 M, 5.0 ml) + 5FU (0.01 M, 5.0 ml) + metal solution (0.01 M, 5.0 ml) + water; (D) $HNO_3$ (0.02 M, 10.0 ml) + $NaNO_3$ (1.0 M, 5.0 ml) + Hm (0.01 M, 5.0 ml) + water; (E) $HNO_3$ (0.02 M, 10.0 ml) + $NaNO_3$ (1.0 M, 5.0 ml) + Hm (0.01 M, 5.0 ml) + metal solution (0.01 M, 5.0 ml) + water; (F) $HNO_3$ (0.02 M, 10.0 ml) + $NaNO_3$ (1.0 M, 5.0 ml) + 5FU (0.01 M, 5.0 ml) + metal solution (0.01 M, 5.0 ml) + Hm (0.01 M, 5.0 ml) + water. In each case, the total volume was maintained at 50.0 ml and ionic strength 0.1 M ($NaNO_3$).

**Preparation of the complexes**

Solution of (1m mole) metal nitrates in 15 ml ethanol and 5FU (1m mole) in 30 ml ethanol were obtained by heating. Both the warm solutions were mixed and the volume of the resultant mixture was reduced to about 50% by heating with constant stirring. The precipitates were obtained at ~ pH 8 by adding aqueous sodium hydroxide solution. Keeping the precipitates in an ice bath and on adding an aqueous solution of (1m mole) histamine to it, a clear solution was obtained. The solid complexes were obtained by concentrating the above solution at ~ 60 °C to 5 ml and on adding diethyl ether. The precipitate was filtered washed with absolute ethanol several times, finally with anhydrous diethyl ether and dried at ~ 50 °C.

The analysis of C, H and N were carried on Perkin-Elmer model 240C elemental analyzer. The metal ions were determined by dissolving the complexes in dilute nitric acid and titrating against EDTA [23]. The infrared spectra of the complexes were registered on a Perkin-Elmer 783 spectrophotometer. The electronic spectra of complexes were registered in the solid state with a Perkin-Elmer Lambda 35 UV/VIS spectrophotometer in the range of 200-1100 nm. Magnetic susceptibility measurements at 305.5 K were done by Faraday magnetic susceptibility balance and X-ray powder data were obtained on Philips PW 1710 diffractometer using Cu-Kα radiation.

**Antitumor activity evaluation**

The antitumour activity both *in vivo* and *in vitro* of the mixed ligand complexes has been evaluated according to the method reported elsewhere [24]. The antitumour response was also measured as median survival time (days) in which median life span was determined and the percentage of increased life span (ILS) was calculated as

$$\% \text{ of ILS} = \left( \frac{\text{median survival time (days) of treated group}}{\text{median survival time (days) of control group}} - 1 \right) \times 100$$

According to the National Cancer Institute [25], the criterion for a significant therapeutic effect is ≥ 30 % ILS for P388 leukemia (ip).

**ACKNOWLEDGMENTS**

Authors are grateful to UGC, New Delhi for the financial assistance.

**REFERENCES**

1. Hiedelberger, C.; Chaudhuri, N. K.; Danneberg, P.; Mooren, D.; Grelsbach, L.; Duschinsky, R.; Schnitzer, R. J.; Pleven, E.; Scheiner, J. *Nature*, **179**, 663, 1957.
2. Fox, J. J.; Wempen, I. *J. Med. Chem.*, **9**, 101, 1966.
3. Rosenberg, B.; Van Camp, L. *Cancer Res.*, **30**, 1799, 1970.
4. Joshi, K. C. *J. Indian Chem. Soc.*, **63**, 187, 1986.
5. (a) Singh, U. P.; Ghose, R.; Ghose, A. K. *Inorg. Chim. Acta*, **136**, 21, 1987. (b) Singh, U. P.; Ghose, R.; Ghose, A. K. *Indian J. Cancer Chemotherapy*, **10**, 147, 1988; **13**, 33, 1991. (c) Singh, U. P.; Ghose, R.; Ghose, A. K.; Sodhi, A.; Singh, S. M.; Singh, R. K. *J. Inorg. Biochem.*, **37**, 325, 1989.
6. Singh, S.; Ghose, A. K. *J. Indian Chem. Soc.*, **73**, 650, 1996.
7. (a) Fernandes, M.; Maria, C.; Paniago, E. B.; Carvalho, S. *J. Braz. Chem. Soc.*, **8**, 537, 1997. (b) Torok, I.; Tamas, G.; Bela, G.; Toth, G. K.; Peter, A. *J. Chem. Soc., Dalton Trans.*, 1205, 1998. (c) Drozdzewski, P.; Kordon, E. *Spectrochim. Acta, Part A*, **56**, 1299, 2000. (d) Szabo-Planka, T.; Rockenbauer, A.; Korecz, L; Nagy, D. *Polyhedron*, **19**, 1123, 2000.
8. (a) Bjerrum, J. Metal Ammine Complex Formation in Aqueous Solution, Haase, Copenhagen, 1941. (b) Irving, H. M.; Rossotti, H. S. *J. Chem. Soc.,* 3397, 1953; 2904, 1954. (c) Chidambaram M. V.; Bhattacharya, P. K. *J. Inorg. Nucl. Chem.*, **32**, 3271, 1970.
9. Motekaitis, R. J.; Martell, A. E. Determination and Use of Stability Constants, VCH Publishers, New York, 1989.
10. Lee, J. D. Concise Inorganic Chemistry, 4th Edn., Chapman and Hall Ltd, London, 1991.
11. Larson, L., *Acta Chem. Scand.*, **4**, 27, 1950.
12. Itabashi, M.; Shoji, K.; Itoh, K. *Inorg. Chem.*, **21**, 3484, 1982.
13. Boudreav, P. A.; Hopper, R. J. *J. Inorg. Nucl. Chem.*, **39**, 1247, 1977.
14. Eilbeck, W. J.; Holmes, F.; Phillips, G. G.; Underhill, A. E. J. Am. Chem. Soc., **89**, 1161, 1967.
15. Ghose, R. *Inorg. Chim. Acta*, **156**, 303, 1989.
16. Goodgame, M.; Johns, K. W. *J. Chem. Soc. Dalton*, 1294, 1978.
17. Speca, A. N.; Mikulski, C. M.; Iaconianni, F. J.; Pytteuski L. L.; Karayannis, N. M. *Inorg. Chim. Acta*, **37**, L551, 1979.
18. Nakamoto, K. Infrared spectra of Inorganic and Coordination Compounds, 2nd edition, Wiley Interscience, New York, 1970.
19. Lever, A. B. P. Inorganic Electronic Spectroscopy, Elsevier, Amsterdam, 1968.
20. Cotton F. A.; Wilkinson, G. Advanced Inorganic Chemistry, 5th edition, Wiley Interscience, New York, 1988.
21. Martin, J. W. L.; Timmons, J. H.; Martell, A. E.; Willis, C. J. *Inorg. Chem.*, **19**, 2328, 1980.
22. Azaroff, L. V. Elements of X-ray Crystallography, New York, 1964.
23. Flaschka, H. A. EDTA Titration, Pergamon, 1964.
24. Singh, U. P.; Ghose, R.; Ghose, A. K.; Singh, R. K.; Sodhi, A.; Geeta, B. *Indian J. Cancer Chemother.,* **13**, 45, 1991.
25. Geran, R. E.; Greenberg, N. H.; MacDonald, M. M.; Schumaker, A. M.; Abbott, B. *J. Cancer Chemother. Rep.*, **3**, 1, 1972.

**Received: May 15, 2002 – Accepted: May 29, 2002 – Accepted in publishable format: May 30, 2002**

